# The influence of participation on mortality in very old age among community-living people in Sweden

**DOI:** 10.1007/s40520-018-0947-4

**Published:** 2018-04-20

**Authors:** Maria Haak, Charlotte Löfqvist, Susann Ullén, Vibeke Horstmann, Susanne Iwarsson

**Affiliations:** 1 Department of Health Sciences, Lund University, Box 157, 221 00 Lund, Sweden; 2 Clinical Studies Sweden – Forum South, Skåne University Hospital, Lund, Sweden

**Keywords:** Activities of daily living, Leisure activity, Longitudinal design, Physical activity, Social engagement

## Abstract

**Background:**

Participation in everyday life and society is generally seen as essential for health-related outcomes and acknowledged to affect older people’s well-being.

**Aims:**

To investigate if aspects of performance- and togetherness-related participation influence on mortality among very old single living people in Sweden.

**Methods:**

ENABLE-AGE Survey Study data involving single-living participants in Sweden (*N* = 314, aged 81–91 years), followed over 10 years were used. Multivariate Cox regression models adjusted for demographic and health-related variables were used to analyse specific items influencing mortality.

**Results:**

Participation in performance- or togetherness-oriented activities was found to significantly influence mortality [HR 0.62 (0.44–0.88), *P* value 0.006, and HR 0.72 (0.53–0.97), *P* value 0.031, respectively]. Talking to neighbours and following local politics had a protective effect on mortality, speaking to relatives on the phone (CI 1.10–2.02) and performing leisure activities together with others (CI 1.10–2.00) had the opposite influence. That is, those performing the latter activities were significantly more likely to die earlier.

**Discussion:**

The main contribution of this study is the facet of the results showing that aspects of performance- and togetherness-related participation have a protective effect on mortality in very old age. This is important knowledge for designing health promotion and preventive efforts for the ageing population. Moreover, it constitutes a contribution to the development of instruments capturing aspects of participation influencing on mortality.

**Conclusion:**

In the development of health promotion and preventive efforts the inclusion of participation facets could be considered in favour of potential positive influences on longevity.

## Introduction

Participation in everyday life and society is generally seen as essential for health-related outcomes and acknowledged to affect older people’s well-being [1]. As participation is one of the core components of the International Classification of Functioning, Disability and Health (ICF) introduced by the World Health Organisation (WHO) [2] and related to functioning and health studying to what extent this component influences mortality deserves more interest.

During recent years, participation has gained increased attention in research on ageing, but little research is available regarding the relation of participation and mortality among community-living very old people. Only few empirical studies focusing on participation among very old community-living people have been published. Most recent studies focus on very old people’s own experience of participation. For example, Hedman et al. [3] investigated the meaning of autonomy and participation when living with chronic illness. Moreover, participation has been shown to prevent isolation and be beneficial in continuing living an independent life at home [4]. As to the relation between participation and mortality, a majority of studies include large samples, but do not differentiate based on age. In one of the few longitudinal studies focusing on those aged 85 years or older, Rizzuto et al. [5] reported from a study in Sweden that aspects of participation are related to longevity. In another study from Sweden including people aged 77 years, followed over 4 years, it was found that participation in solitary-active activities significantly reduced mortality [6]. In the North American Alameda County Study, 6157 people were followed from the age of 21 and 35 years on. Having three or more close friends, regularity of church attendance and participation in social/recreational groups were among the factors that significantly decreased mortality [7]. Applying another perspective, a concept such as social isolation could be studied as an antithesis to participation. For example, following 6500 people in England aged 52 years or older during 7.5 years, Steptoe et al. [8] found that social isolation was significantly associated with higher mortality. Summing up on this, while the definitions and operationalization of participation differ among studies, there is some evidence that participation is related to mortality, but regarding those living into very old age there is a need for more research.

Participation is a broad concept of interest to researchers in many disciplines, yet there is no consensus in the literature regarding its definition and operationalization see e.g., [1]. There are many related concepts such as social capital [9], social engagement [10], and social integration [11], and those are all to some extent overlapping with participation. Influenced by the biopsychosocial model ICF [2], participation is most often defined from an individual perspective as “the person’s involvement in a life situation”, which could be either with a focus on the execution of an activity, on the interaction with others or just simply to be involved in community life. The ICF definition of participation is used as a starting point for our study.

In a previous qualitative study, very old community-living people in urban districts were interviewed in depth about how they experienced participation in relation to their home and neighbourhood environments [12]. Two dimensions of participation emerged from the interviews; togetherness-oriented and performance-oriented participation. Togetherness-oriented participation includes sharing experiences with others through communication, but togetherness was also illustrated by just being in a familiar context or being surrounded by people symbolising some kind of togetherness. It could also be about experiencing a sense of participation without interaction with others. For example, by following news and media participants expressed a sense of being part of a larger context.

Performance of activities constituted a medium through which participation was experienced, with the performance as such at the centre. It could be about performing for the purpose of helping others, being important for others and/or taking responsibility, which created a feeling of still being able to contribute with knowledge and skills. Performance-oriented participation was also about performing with personal satisfaction. Lack of satisfaction in performing activities was conducive to reduced participation.

As part of a subsequent study we demonstrated the validity of the two dimensions of participation using quantitative data from the same project [13]. To validate the two dimensions, experts independently scrutinised a survey study questionnaire based on well-established assessments from psychology, gerontology, geriatrics, and social sciences to identify items that reflected togetherness-oriented and performance-oriented participation as defined in the qualitative study [12], respectively. The fact that there were medium significant correlations between the rank scores for the two dimensions indicates that they are related but different. Thus, this process ended up in a set of validated items defining the two dimension of participation used in this study.

As yet, participation has not been studied in relation to mortality with such dimensions of in mind. Accordingly, the purpose of the present study was to investigate if aspects of performance-and togetherness-related participation influenced mortality among very old single living people in Sweden.

## Methods

### The ENABLE-AGE project context

This study was based on Swedish data from the longitudinal survey study of the cross-national European project “Enabling Autonomy, Participation, and Well-Being in Old Age: The Home Environment as a Determinant for Healthy Ageing” (ENABLE-AGE) [14]. The project design was explicitly explorative, with the main objective to examine the home environment and its importance for autonomy, participation and well-being among very old people living in single-person households in urban areas. All participants were enrolled after informed consent; the Swedish part of the ENABLE-AGE Survey Study was approved by the Ethics Committee at Lund University, Sweden. Details regarding the project design and methodology have been published elsewhere, see e.g., [14].

### Sample description

#### Study sample

The target population was very old persons living alone in ordinary housing in three geographically defined urban areas in southern Sweden. The study sample was drawn at random from the Swedish Population Register, stratified for age and sex. That is, the intention was to obtain 50% of the sample within the age span 80–84 years and the remaining 50% in the age span 85–90 years, and to obtain 75% women and 25% men. Out of 970 persons contacted 41% accepted to participate; the final sample consisted of 397 persons. The most common reasons for not participating were poor health (27%) and lack of time and interest.

Data were collected at home visits by trained interviewers for the first time in 2002–2003, with a follow-up 1 year later. At this follow-up, questions addressing different aspects of participation were included. As participation is the key issue in the present study, our analyses were based on the participants taking part in the 1-year follow-up. At the 1-year follow-up, 73 participants had dropped out; 16 of those were dead. Thus, 314 participants were included in the present study. Data on mortality were retrieved from the Swedish Central Population Register 10 years after the follow-up, with information about exact date of death for the deceased. A detailed description of the study sample is presented in Table 1.

**Table 1:** Description of the study sample (*N* = 314)

Variable	2003–2004 *N* = 314
Age median (q1, q3)	85.6 (82.4, 88.2)
Sex, *n* (%)	
Men	82 (26.1)
Women	232 (73.9)
Civil status, *n* (%)	
Divorced	24 (7.6)
Widowed	253 (80.6)
Never married	37 (11.8)
Monthly income, euro	
≤ 900	145 (52.0)
>900	134 (48.0)
Perceived health, *n* (%)	
Poor/fair	112 (35.7)
Good	110 (35.0)
Very good	61 (19.4)
Excellent	31 (9.9)
No. of symptoms, median (q1, q3)	9 (4.75, 12.0)
Functional limitations^a^	
No	69 (22.0)
Yes	245 (78.0)
Dependence on mobility devices^a^, *n* (%)	
No	158 (50.3)
Yes	156 (49.7)
P-ADL independence, *n* (%)	
0	2 (0.6)
1	1 (0.3)
2	2 (0.6)
3	7 (2.2)
4	32 (10.2)
5	270 (86.0)

q1—1st quartile; q3—3rd quartile. P-ADL—number of items performed independently. No. of symptoms: out of 33. Perceived health: scored from 1 = Poor to 5 = Excellent

^a^According to the Housing Enabler [27]

### Instruments

Besides questions related to participation, the comprehensive ENABLE-AGE Survey Study Questionnaire incorporated a wide range of questions on demographics, self-report scales and observational assessments. The demographic information used in the present study included data on age, sex, civil status, income, perceived health, number of symptoms, number of functional limitations, dependence on mobility devices and P-ADL independence. Data on aspects of health and participation were included in the analyses; the instruments used for these variables are described below.

#### Health-related variables

Self-ratings of *perceived health* were collected by means of the well-established question from the SF-36 questionnaire “In general would you say your health is?” with five response alternatives (from 1 = excellent to 5 = poor) [15]. The total *number of self-reported symptoms* was calculated for each participant. From a list of 33 symptoms listed, 30 from Tibblin et al. [16] plus three study specific symptoms concerning incontinence and dental problems, each participant selected those that were present during the last month. The total *number of functional limitations* (13 items) and *dependence on mobility devices* (2 items) from the Housing Enabler instrument [17] were calculated. *Activities of daily living* (ADL) were assessed using the ADL Staircase [18], administered by a combination of interview and observation regarding five personal ADL (P-ADL) items (feeding, transferring, going to the toilet, dressing, bathing) and four instrumental ADL (I-ADL) items (cooking, shopping, cleaning, and transportation). Each item was assessed on a 3-graded scale: independent, partly dependent, or dependent, where dependence indicated that the person actually received personal assistance. The variable *number of P-ADL performed independently* was constructed and in this study considered to be a health variable, while I-ADL was considered a participation-related variable. *Cognitive functioning* was assessed using five items from the Mini Mental State Examination (MMSE) [19, 20] using the proportion of correct performed tasks. A *depression symptom sum score* was obtained from the 15-item version of the Geriatric Depression Scale [21].

#### Participation-related variables

Based on our previous study [13] we constructed variables representing togetherness-oriented participation and performance-oriented participation, respectively. Thus, a set of variables related to performance-oriented participation was used (see Table 2). The variable *number of I-ADL performed independently* was constructed based on ADL Staircase [18] data (constructed as described above under health-related variables). The self-rated question *In general, how often do you go out these days?* was assessed on a five point scale ranging from 1 (every day) to 5 (never). *Perceived functional independence* was assessed on a scale ranging from 0 (completely dependent) to 10 (completely independent). Further, each participant was asked to list up to three leisure time activities performed indoors and three activities performed outdoors; for each activity it was registered whether it was performed alone or together with others. Thus, the variable *number of leisure activities performed alone* was constructed (range 0–6). In addition, there were three questions related to the *frequency of performance-oriented participation in social activities*, each with five response options (i.e., every day, one/two times a week, one/two times a month, one/two times a year, almost never).

**Table 2:** Participation-related variables

Performance-oriented participation		Togetherness-oriented participation	
Variable *n* (%)	2003–2004 *N* = 314	Variable *n* (%)	2003–2004 *N* = 314
How often do you go out^a^	224 (71.6)	Visit friends^c^	189 (60.4)
I-ADL independence^e^	196 (62.4)	Speak to friends on phone^a^	220 (70.3)
Perceived independence^f^	134 (43.2)	Visit relatives^c^	207 (67.0)
No. of leisure activities alone^g^	163 (51.9)	Speak to relatives on phone^a^	129 (41.2)
Helping others^d^	138 (44.2)	Receive visits^b^	169 (54.3)
Fitness^a^	175 (56.1)	Speak to neighbors^b^	217 (70.0)
Volontary groups^d^	70 (22.4)	Engage in leisure^c^	182 (58.3)
		Participate in organised activities^d^	101 (32.4)
		Talk about politics^d^	136 (43.7)
		Follow local politics^a^	211 (67.6)
		Attend adult class^d^	41 (13.1)
		Leisure activities with others^h^	130 (41.4)

^a^Every day

^b^Once/twice a week or more often

^c^Once/twice a month or more often

^d^Once/twice a year or more often

^e^Independent in 3 or more out of 4

^f^Completely independent

^g^Participating in 4 or more activities

^h^2 or more. *N* varied between 310 and 314

For togetherness-oriented participation the variable *number of leisure activities performed with others* was constructed in the same way as above (range 0–6). Moreover, there were eleven questions related to the *frequency of togetherness-oriented participation in social activities*, rated and dichotomised as described above.

### Statistics

Data were described with numbers and percentages or median and quartiles. All the individual underlying variables selected to be included in the two subsequently summed participation-related variables were dichotomized by the median (less often/more often). Therefore, the cut off between less often and more often differed among the variables, not only due to the different scales used, but also because some were in general more frequently performed than others. Then sum scores for performance-oriented and togetherness-oriented participation variables, respectively, were created in a two-step procedure using the dichotomised underlying activity variables. In the first step (for each of the two summed participation variables), for each participant we counted the number of the individual underlying activity variables that were performed more often. For both summed participation scores, this resulted in scores between 0–7 and 0–12, respectively, where 0 corresponds to the participant performing none of the underlying activities more often, and 7 or 12 corresponds to the participants performing all those activities more often. In the second step, the scores from the first step were dichotomised by the median to distinguish between participants with ‘few underlying activities that were performed more often’ and those with ‘higher number of underlying activities that were performed more often’.

The two sum scores were analysed using Cox regression adjusted for demographic and health-related variables. In the next step, analysing which specific items that influenced mortality one Cox regression model for the variables who belong to performance-oriented participation and one for variables belonging to togetherness-oriented participation was performed. In both multivariable models we adjusted for demographic and health-related variables, and when analysing togetherness-oriented participation we also included the sum score for participation and vice versa. Since the number of variables was too large in relation to the number of events (i.e., deaths) we reduced the models stepwise by excluding the variable with the largest *P* value. This reduction continued until all *P* values were below 0.15 or until some of the estimates changed by more than 10%.

## Results

Participation in performance-oriented or togetherness-oriented activities was found to be significantly associated with mortality. The Hazard Ratio (HR) for performance-oriented sum score was 0.62 (0.44–0.87), *P* value 0.006, and HR for togetherness-oriented sum score was 0.72 (0.53–0.97), *P* value 0.031 (see Table 3). That is, participants with a higher number of performance- and togetherness-oriented activities reported to be performed more often were less likely to die early compared to those reporting fewer performance- and togetherness-oriented activities performed more often.

**Table 3:** Aspects of performance-oriented and togetherness-oriented participation and their relation to mortality in very old age

Variable	HR	95% confidence interval	*P* value
Performance-oriented participation	0.62	(0.44, 0.88)	0.006
Togetherness-oriented participation	0.72	(0.53, 0.97)	0.031
Performance-oriented participation			
Helping others	0.73	(0.54, 0.98)	0.040
Participate in voluntary groups	1.34	(0.94, 1.92)	0.104
No. of leisure activities performed alone	0.60	(0.45, 0.81)	0.001
I-ADL independence	0.59	(0.42, 0.83)	0.002
Togetherness-oriented participation			
Visit friends	0.77	(0.56, 1.07)	0.124
Speak to friends on the phone	0.79	(0.56, 1.11)	0.178
Visit relatives	0.76	(0.55, 1.06)	0.105
Speak to relatives on the phone	1.49	(1.10, 2.02)	0.010
Speak to neighbours	0.70	(0.52, 0.96)	0.026
Follow local politics	0.48	(0.35,0.67)	0.000
No. of leisure activities with others	1.49	(1.10, 2.00)	0.009

Statistical significance at *P* value < 0.05

*HR* hazard ratios

Concerning aspects of performance-oriented participation, those who were helping others (CI 0.54–0.99), performed leisure activities alone (CI 0.45–0.81) and were independent in I-ADL (CI 0.42–0.83) were less likely to die earlier.

As to aspects of togetherness-oriented participation that significantly influencing mortality constituted the following variables; speaking to relatives on the phone, speaking to neighbours, following local politics, and performance of leisure activities performed together with others. These aspects, however, influenced mortality in opposite directions. That is, talking to neighbours and following local politics had a protective effect on mortality, while speaking to relatives on the phone (CI 1.10–2.02) and performing leisure activities together with others (CI 1.10–2.00) had the opposite influence. That is, those performing the latter activities were significantly more likely to die earlier.

## Discussion

The main contribution of this study is the facet of the results showing that aspects of performance- and togetherness-related participation have a protective effect on mortality in very old age. This is important knowledge for designing health promotion and preventive efforts for the ageing population. Moreover, it constitutes a contribution to the development of instruments capturing aspects of participation influencing on mortality.

Due to the fact that the results were obtained controlling for several objective and perceived aspects of health, the study shows that independence in more complex activity performance (I-ADL independence) as well as helping others and being able to perform leisure activities alone (number of leisure activities alone) contribute to the longevity among those living in the community into very old age.

On an overarching level, the present study is well in line with previous research in this field. That is, independent performance of daily life activities and social activities are associated with longevity [22], and this has been shown not the least in other longitudinal studies in Sweden. For example, Rizzuto et al. [5] showed that very old people with a low risk profile in terms of healthy lifestyle behaviours, a rich or moderate social network and participation in at least one leisure time activity survived 4 years longer than those with a high-risk profile did.

In addition, our study shows that different facets of participation, are working in opposite directions regarding mortality. More specific, as we have more detailed and specified data on participation our findings indicate that taking part in a number of leisure activities together with others, probably in the sense that you need support from another person, implies a higher mortality risk. This also goes for those speaking with relatives on the phone, presumably indicating feelings of a worrying or insecure situation or being in need of assistance. This kind of new detailed knowledge is important as society is striving for inclusion and equality along the process of ageing [23]. However, as the scores on individual activity variables underlying the summed togetherness-oriented participation variables pointed in different directions, the validity of that sum score could be questioned and considered as a study limitation. Variables not reflecting togetherness in a coherent direction could be considered for removal from the summed score. Making this change and computing the results again using our data, the HR for the sum score of togetherness-oriented participation decreased, which implies an even stronger association with mortality. Applying a critical stance to the results of the present study, an alternative would be to include this adjustment of the summed participation variables and account for the results of these post hoc analyses in “Results” section. However, rather than presenting such data driven results, we suggest further validation of the summed participation scores in future studies, in other samples.

At the time for the data collection very few instruments were readily at hand specifically targeted participation. One instrument available was the Impact on Participation and Autonomy (IPA) questionnaire [24], but with a focus on work and education it could not be considered valid for use with very old people. Other examples are the LIFE-H [25] and the Participation Survey/Mobility, PARTS/M, [26], but they were both published after the inception of the ENABLE-AGE project.

Given the lack of consensus as regards the conceptual definition of participation, and the shortage of instruments targeting participation for ageing people, for the present study we instead started out using two dimensions hitherto not studied outside the ENABLE-AGE Project. The dimensions performance- and togetherness-related participation were identified based on a qualitative study [12] followed by a quantitative study confirming them in the same sample [13], that is, the Swedish part of the ENABLE-AGE project. Accordingly, studying these dimensions of participation as related to mortality in the same sample is highly valid, but their validity for studies in other samples remains to be confirmed. In this study, we grouped and used several variables within the two dimensions of participation in multivariable analysis. The findings demonstrate that making use of these variables was a successful and nuanced way to increase the understanding of the complex participation construct. Our findings also contribute with variables capturing the influence of mortality in both directions; aspects that enhance longevity as well as aspect indicating an earlier death. Thus, these findings constitute an important knowledge contribution to the creation of new instruments aiming to capture performance-related and togetherness-related participation aspects for the ageing population.

It could be argued that good health is a prerequisite for participation, and thus these results would merely be an indication that healthy people can participate in life situations. However, in this study aiming to investigate if aspects of participation influenced mortality in this relatively healthy sample, the fact that they were still in this very high age able to participate in everyday life and society add some strength to this study. It should, however, be kept in mind that studying other samples of ageing people could reveal other variables influencing mortality.

## Conclusion

Participation in performance-oriented or togetherness-oriented activities significantly influences mortality in very old age. In the development of health promotion and preventive efforts the inclusion of participation facets could be considered in favour of potential positive influences on longevity.
